# Accumulation of dibenzocyclooctadiene lignans in agar cultures and in stationary and agitated liquid cultures of *Schisandra chinensis* (Turcz.) Baill

**DOI:** 10.1007/s00253-015-7230-9

**Published:** 2015-12-21

**Authors:** Agnieszka Szopa, Adam Kokotkiewicz, Urszula Marzec-Wróblewska, Adam Bucinski, Maria Luczkiewicz, Halina Ekiert

**Affiliations:** Chair and Department of Pharmaceutical Botany, Collegium Medicum, Jagiellonian University, ul. Medyczna 9, 30-688 Kraków, Poland; Chair and Department of Pharmacognosy, Faculty of Pharmacy, Medical University of Gdansk, al. gen. J. Hallera 107, 80-416 Gdańsk, Poland; Department of Biopharmacy, Faculty of Pharmacy, Ludwik Rydygier Collegium Medicum in Bydgoszcz, Nicolaus Copernicus University in Toruń, ul. dr A. Jurasza 2, 85-089 Bydgoszcz, Poland

**Keywords:** Chinese magnolia vine, Schizandra, Schisandra lignans, In vitro culture, Shoot-differentiating culture, Shoot culture

## Abstract

*Schisandra chinensis* plant in vitro cultures were maintained on Murashige and Skoog (MS) medium supplemented with 3 mg/l 6-benzyladenine (BA) and 1 mg/l 1-naphthaleneacetic acid (NAA) in an agar system and also in two different liquid systems: stationary and agitated. Liquid cultures were grown in batch (30 and 60 days) and fed-batch modes. In the methanolic extracts from lyophilized biomasses and in the media, quantification of fourteen dibenzocyclooctadiene lignans identified based on co-chromatography with authentic standards using high-performance liquid chromatography with diode array detection (HPLC-DAD) and/or liquid chromatography with diode array detection and electrospray ionization mass spectrometry (LC-DAD-ESI-MS) methods. For comparison purposes, phytochemical analyses were performed of lignans in the leaves and fruits of the parent plant. The main lignans detected in the biomass extracts from all the tested systems were schisandrin (max. 65.62 mg/100 g dry weight (DW)), angeloyl-/tigloylgomisin Q (max. 49.73 mg/100 g DW), deoxyschisandrin (max. 43.65 mg/100 g DW), and gomisin A (max. 34.36 mg/100 g DW). The highest total amounts of lignans in the two tested stationary systems were found in extracts from the biomass harvested after 30 days of batch cultivation: 237.86 mg/100 g DW and 274.65 mg/100 g DW, respectively. In the agitated culture, the total content reached a maximum value of 244.80 mg/100 g DW after 60 days of the fed-batch mode of cultivation. The lignans were not detected in the media. This is the first report which documents the potential usefulness of *S. chinensis* shoot cultures cultivated in liquid systems for practical purposes.

**Electronic supplementary material:**

The online version of this article (doi:10.1007/s00253-015-7230-9) contains supplementary material, which is available to authorized users.

## Introduction

*Schisandra chinensis* (Turcz.) Baill., Chinese magnolia vine (*Schisandraceae*), is a valuable medicinal plant species that has long been used in traditional and modern Far Eastern medicine (Szopa and Ekiert 2014). It has also gained a very important position in modern North American (United States Pharmacopeia 1999) and European phytotherapy (European Pharmacopoeia 8.0 2013). Since 2007, this plant species has been listed as an important medicinal plant in the International Pharmacopoeia edited by WHO (2007).

Schisandra fruit extracts show valuable biological activities, such as hepatoprotective, anticancer, adaptogenic, antioxidant, and anti-inflammatory properties (Hancke et al. 1999). All these medicinal qualities are primarily attributed to dibenzocyclooctadiene lignans, known even in the professional scientific literature as “schisandra lignans” (WHO Monographs on Selected Medicinal Plants 2007). According to the data contained in the monograph on *Fructus Schisandrae* in the International Pharmacopoeia (2007), the number of isolated and identified lignans in fruit extracts is about thirty. These compounds usually have several synonymous names. The main compounds are schisandrin, schisandrin B, schisandrin C, schisantherin A, schisantherin B, deoxyschisandrin, *γ*-schisandrin, gomisin A, and gomisin G (Opletal et al. 2004; Szopa and Ekiert 2014; WHO Monographs on Selected Medicinal Plants 2007).

Chemical synthesis of this group of lignans is theoretically possible, but it is a multistage and very complicated process (Chang et al. 2005). There is only one synthetic drug available in therapy, diphenyldimethylbicarboxylate (DDB), a derivative of schisandrin C (Ip et al. 2000). The natural plant raw material, the schisandra fruits, remains indispensable as the basis for medicines, food supplements, and cosmetics.

The main source of this plant raw material in the European countries, and also in the USA and Japan, is imports from China (Wu et al. 2008).

Valuable potential alternatives are offered by plant biotechnological methods making use of in vitro cultures of *Schisandra chinensis*. The biotechnological possibilities of producing some schisandra lignans in the biomass of agar shoot-differentiating callus cultures maintained in Erlenmeyer flasks had been documented by us earlier (Szopa et al. 2015; Szopa and Ekiert 2011, 2013, 2015). Encouraged by the promising results, we decided to undertake the next steps in an attempt to improve the scale of in vitro culture systems and the production of lignans. For this purpose, agar cultures and stationary liquid cultures in special Magenta^TM^ vessels, and also agitated cultures, were tested.

All the cultures were grown on a variant of Murashige and Skoog (MS) medium (1962) containing 3 mg/l 6-benzyladenine (BA) and 1 mg/l 1-naphthaleneacetic acid (NAA). This variant had proved to be the best universal “growth” and “production” medium in our earlier experiments with agar shoot-differentiating callus cultures of schisandra (Szopa and Ekiert 2011, 2013, 2015).

All the types of cultures were grown in batch systems (30 and 60 days). Stationary and agitated liquid cultures were grown additionally in a fed-batch mode (60 days, with fresh medium supplementation on the 30th day of experiment). In addition, the dynamics of the accumulation of lignans was studied in the agitated cultures. Comprehensive insight into the qualitative and quantitative profile of dibenzocyclooctadiene lignans in the sample extracts was achieved using the high-performance liquid chromatography with diode array detection (HPLC-DAD) and liquid chromatography with diode array detection and electrospray ionization mass spectrometry (LC-DAD-ESI-MS) methods.

## Materials and methods

### Experimental in vitro cultures

The shoot-differentiating callus cultures of *Schisandra chinensis* (*Schisandraceae*) were established from leaf buds of plants growing in Rogów Arboretum, Warsaw University of Life Sciences, Forest Experimental Station in Rogów (Poland). Plants were originated from Vladivostok Botanical Garden-Institute, Far East Branch of Russian Academy of Sciences, accession number 13606, germinated in 1993. Plants were taxonomically verified by scientific staff of Rogów Arboretum (Szopa and Ekiert 2011). Experimental agar shoot-differentiating callus cultures and two types of shoot liquid cultures, stationary and agitated (four series), were maintained on Murashige–Skoog (MS) medium supplemented with 3.0 mg/l of cytokinin, 6-benzyladenine (BA), and 1.0 mg/l of auxin, 1-naphthaleneacetic acid (NAA). The cultures were grown under constant artificial light (white fluorescent lamps, 36 W, light intensity 88 ± 8 μmol m^−2^ s^−1^, Philips, Amsterdam, Netherlands), at a temperature of 25 ± 2 °C.

### Agar cultures

**Fig. 1:**
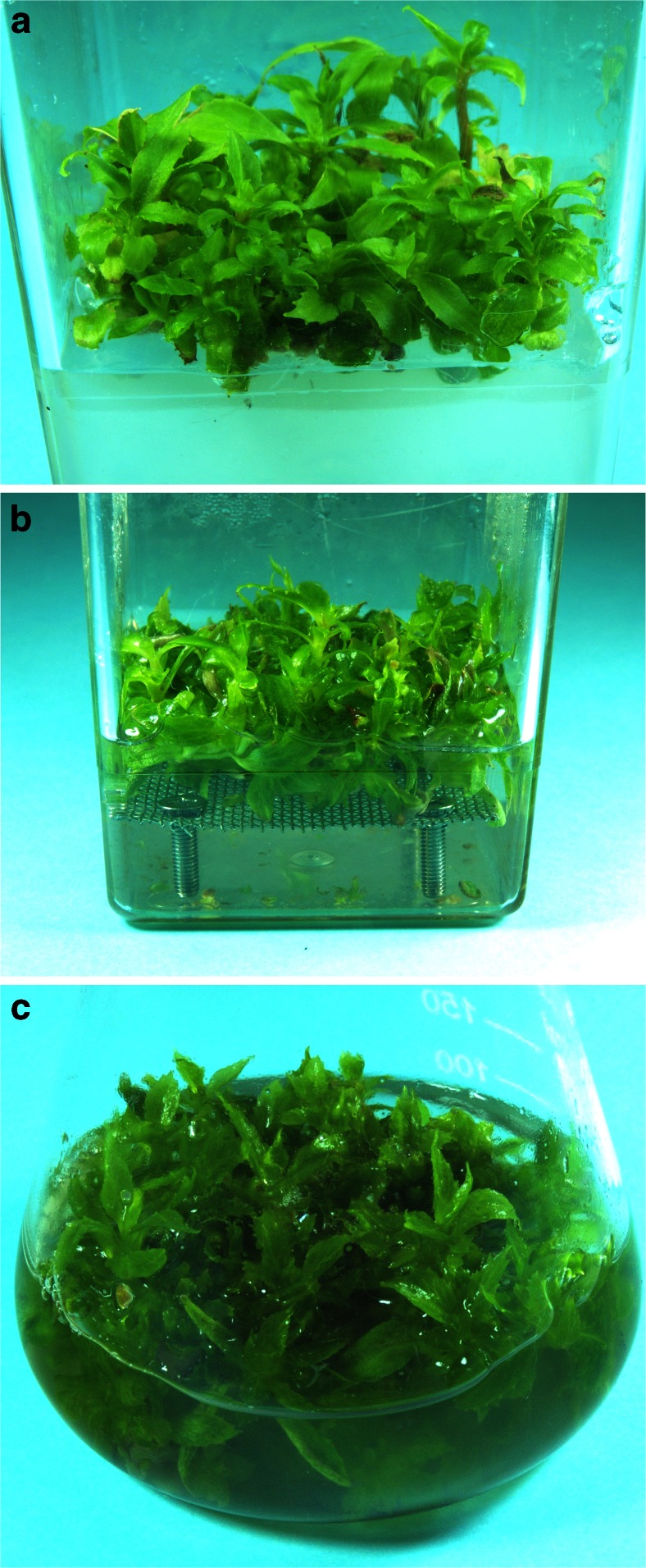
*Schisandra chinensis* biomass from tested in vitro systems: agar cultures (**a**), stationary liquid cultures (**b**), and agitated cultures (**c**)

Agar shoot-differentiating callus cultures were maintained in Magenta^TM^ vessels (77 mm × 77 mm × 97 mm, Sigma-Aldrich, St. Louis, MO, USA; Fig. 1a). For cultivation, 3.0 g of inoculum was introduced into the vessel containing 100 ml of an agar-solidified medium. These cultures were harvested after 30 and 60 days of batch-mode cultivation.

### Stationary liquid cultures

Stationary liquid cultures were maintained in Magenta^TM^ vessels (77 mm × 77 mm × 97 mm,) fitted with stainless steel mesh (1 × 1 mm), placed 13 mm above the bottom, for shoot immobilization (Fig. 1b). For cultivation, 3.0 g of inoculum was introduced into the vessel containing 100 ml of a liquid medium. Cultures of this type were grown in the batch mode (samples were harvested after 30 and 60 days), and additionally in the fed-batch mode (60 days, with fresh medium supplementation, 40 ml, on the 30th day of experiment).

### Agitated cultures

In order to initiate agitated cultures, 3.0 g of inoculum was introduced into a 250 ml Erlenmeyer flask containing 100 ml of a liquid medium (Fig. 1c). The cultures were agitated on a rotary shaker (120 rpm, 25.4 mm orbit, INNOVA 2300, Eppendorf, Hamburg, Gemany). The cultures were grown in the batch mode; the biomass was harvested every 10 days of cultivation for up to 60 days. Additionally, an experiment with the fed-batch mode of cultivation, 60 days, with fresh medium supplementation (40 ml) on the 30th day of experiment, was performed.

### Plant material

The material from the parent plant was harvested in Rogów Arboretum, Warsaw University of Life Sciences, Forest Experimental Station in Rogów (Poland) in 2014 and was analyzed for comparison. It consisted of the leaves and fruits of *Schisandra chinensis*.

### Extraction, HPLC-DAD and LC-DAD-ESI-MS analyses

Dried, lyophilized biomass from in vitro cultures, collected after the growth cycles (four series) and the plant material, leaves and fruits (0.5 g samples of each), was extracted by sonication with 50 ml of methanol two times for 30 min. Experimental, respective media were collected (40 ml) and lyophilized and then were dissolved in methanol (5 ml). In the methanolic extracts, chromatographic quantification of lignans was performed using the HPLC-DAD method developed by Zhang et al. (2009). Separation was performed using a Kinetex^TM^ C-18 analytical column (150 × 4.6 mm, 2.6-μm particle size, Phenomenex, Torrance, CA, USA) at 30 °C. The mobile phase consisted of acetonitrile (A) and water (B), set at a flow rate of 0.8 ml/min. A gradient program was as follows: 0–4 min, 40–45 % B; 4–12 min, 45–50 % B; 12–16 min, 50–68 % B; 16–20 min, 68–75 % B; 20–25 min, 75–95 % B, with a hold time of 15 min, and injection volume 5 μl. Detection wavelength was set at 225 nm. Identification and quantification were made by comparison with nine standards: gomisin A, deoxyschisandrin, schisandrin, and γ-schisandrin (ChromaDex®, Irvine, USA); gomisin G and schisantherin A (PhytoLab GmbH & Co. KG, Vestenbergsgreuth, Germany); and schisandrin C, schisantherin B, and schisanthenol (ChemFaces Biochemical Co. Ltd., Wuhan, People’s Republic of China). Quantification of the tentatively identified compounds (based on LC-DAD-ESI-MS analyses) was performed using the calibration curve for schisandrin, as the main component of the group of dibenzocyclooctadiene lignans.

Qualitative LC-DAD-ESI-MS analyses of schisandra lignans in extracts from the biomass from in vitro cultures and from the plant material (leaves and fruits) were conducted using the Shimadzu chromatographic system (Kyoto, Japan), consisting of two solvent pumps (LC-20 AD), a liquid phase degasser (DGU-20A3), an autosampler (SIL-20 AC, 24 °C), a column oven (CTO-20 AC, 30 °C), a diode array detector (SPD-M20A), and a mass spectrometry detector (2010EV). Separations were performed on Kinetex^TM^ C-18 (150 × 4.6 mm, 2.6-μm particle size, Phenomenex, Torrance, CA, USA) at 0.8 ml/min flow rate. The mobile phase consisted of acetonitrile (A) and water (B). The gradient program was as follows: 0–4 min, 40–45 % B; 4–12 min, 45–50 % B; 12–16 min, 50–68 % B; 16–20 min, 68–75 % B; and 20–25 min, 75–95 % B. The injection volume was 20 μl. LC-DAD data were recorded over the 200–800-nm range. MS spectra were acquired in positive ion mode over the range of *m/z* 50–1000 (scan acquisition). ESI parameters were as follows: detector voltage 1.5 kV, interface temperature 250 °C, CDL (curved desolvation line) temperature 230 °C, heat block temperature 200 °C, and nebulizing gas flow 0.18 l/min.

### Statistical analysis

The obtained results are expressed as the mean ± SD of four independent experiments and determinations.

## Results

### Effect of cultivation mode on biomass growth

In the tested culture systems of *Schisandra chinensis*, we recorded very different Gi values, from about 139 to 1959 %.

**Fig. 2:**
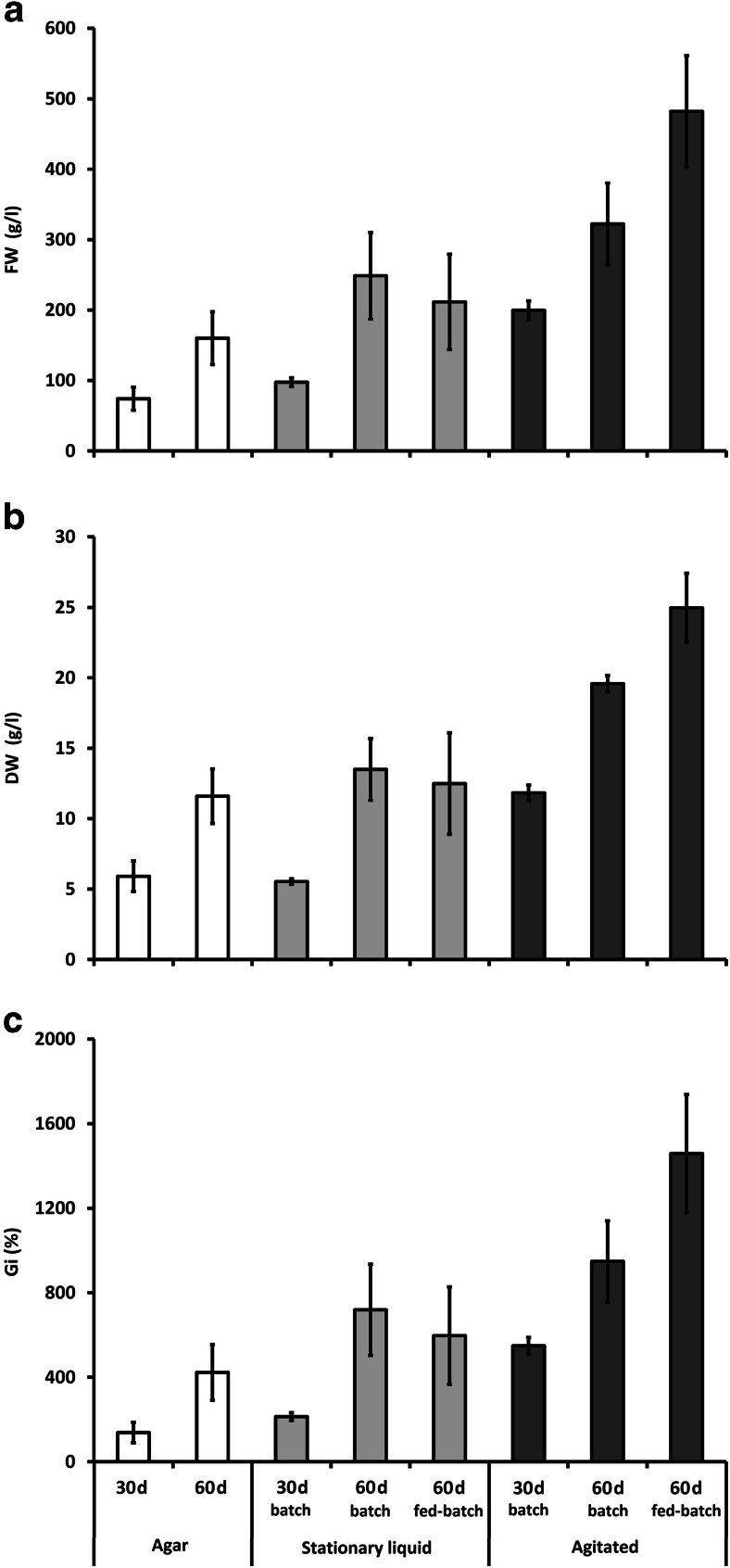
Comparison of growth parameters of biomass from all tested in vitro systems of *Schisandra chinensis* (agar cultures, stationary liquid cultures, and agitated cultures): fresh weight (FW) - **a**, dry weight (DW) - **b**, and growth index (Gi) - **c** after 30 and 60 days of batch and fed-batch mode growth cycles. Values represent the mean ± SD of four samples

The shoots tolerated well the change in the tested system conditions from agar to stationary liquid, and also to agitated culture; neither explant necrosis nor medium browning was observed (Fig. 1). As shown in Fig. 2, the growth parameters (fresh weight, FW; dry weight, DW; and growth index, Gi) of *S. chinensis* shoots maintained in stationary liquid cultures were higher compared with those in the agar shoot-differentiating callus culture; however, the observed differences were less prominent for DW. Agitation clearly promoted shoot culture growth which was reflected by higher FW, DW, and Gi values.

As presented in Fig. 2, the established agitated cultures were characterized by a typical growth cycle with not well-defined death phase.

**Fig. 3:**
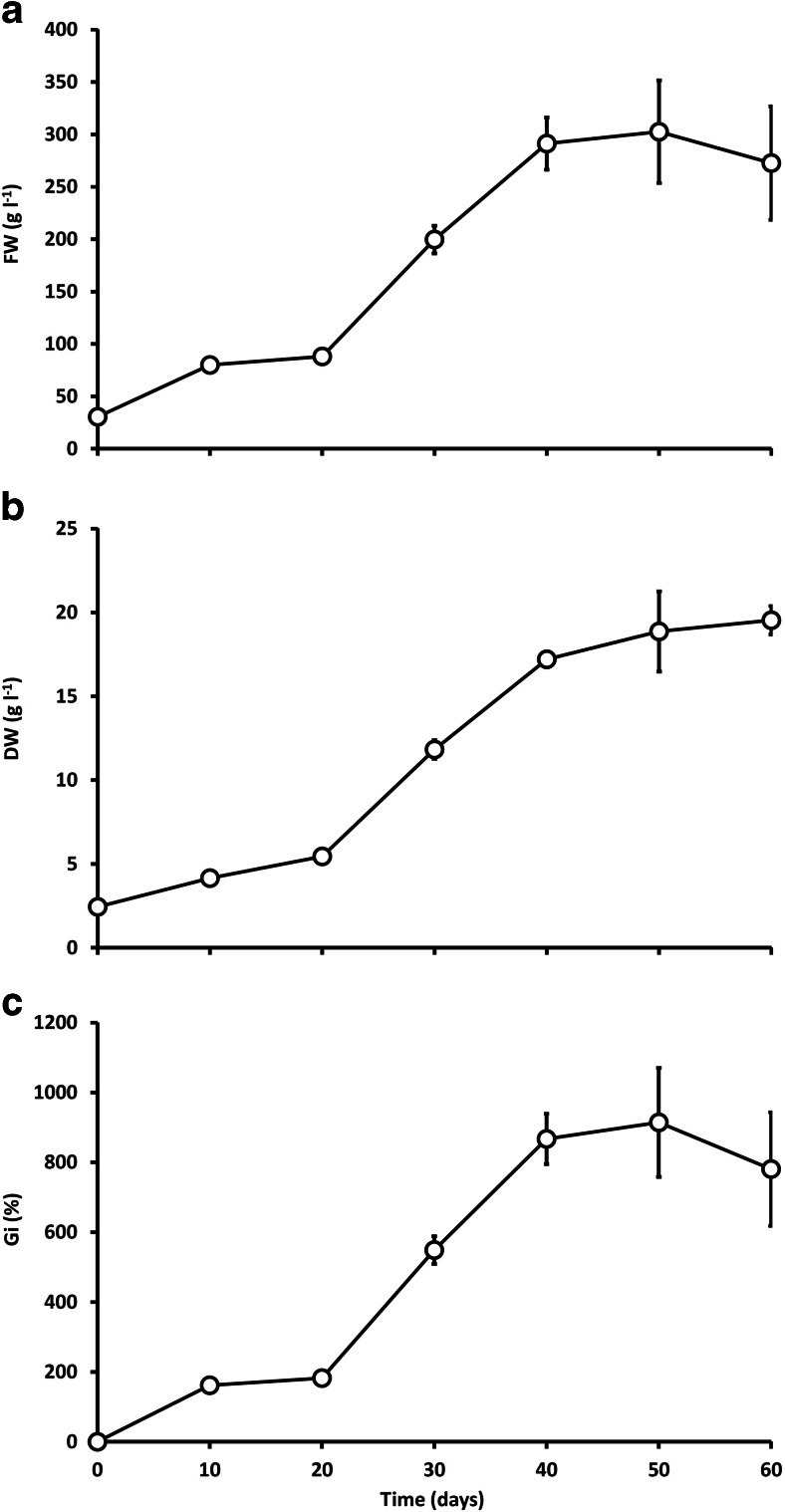
Changes in fresh weight (FW) - **a**, dry weight (DW) - **b**, and growth index (Gi) - **c** of *Schisandra chinensis* biomass from agitated shoot cultures during 60-day growth cycles. Values represent the mean ± SD of four samples

The change of the cultivation mode from batch to fed-batch mode produced positive effects in the agitated cultures, providing 53 and 27 % increases in FW and DW, respectively. On the other hand, the same procedure negatively affected the shoots grown in the stationary liquid system, decreasing the biomass yield of the culture (Fig. 3).

### Analyses of lignans with LC-DAD-ESI-MS

**Fig. 4:**
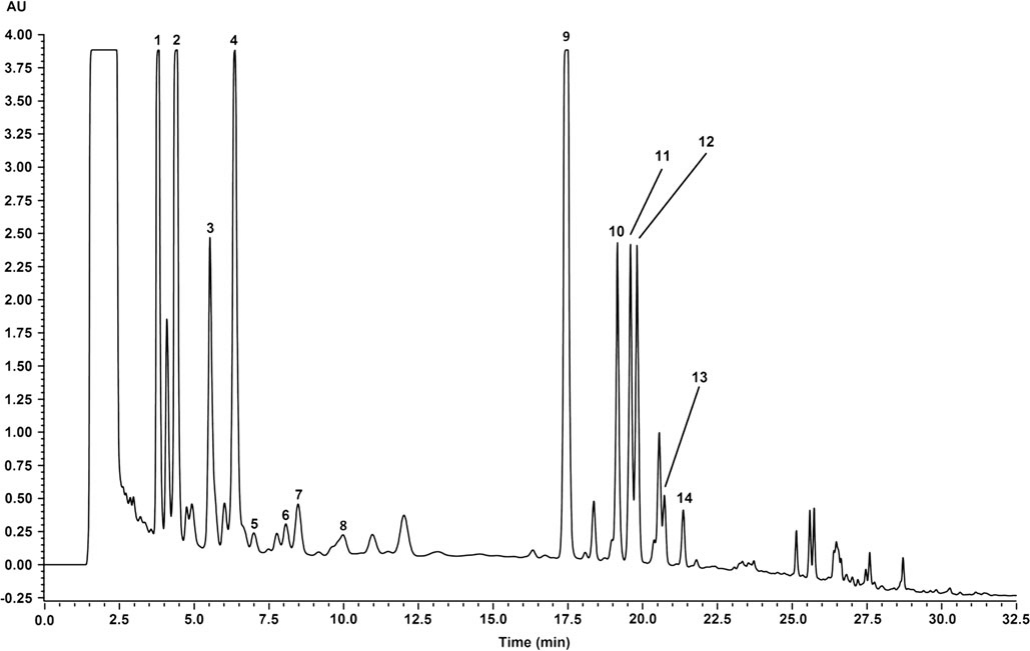
The representative LC-UV chromatogram (λ = 225 nm) of a methanol extract of *Schisandra chinensis* biomass cultivated in vitro. Peak labels correspond to Table 1

Qualitative profiles of lignans from biomass extracts of all the tested culture systems were the same. The representative LC-UV chromatogram of the methanol extract of *Schisandra chinensis* in vitro cultivated biomass is presented in Fig. 4, and the results of LC-DAD-ESI-MS analysis are summarized in Table 1. The UV spectra of peaks 1–14 showed three absorption maxima at approximately 220, 255, and 280 nm (Table 1), characteristic for biphenyl chromophore of dibenzocyclooctadiene lignans (Chang et al. 2005). The identity of compounds 1, 2, 5, 6, 7, 8, 9, 11, and 13 (schisandrin, gomisin A, gomisin G, schisantherin A, schisantherin B, schisanthenol, deoxyschisandrin, *γ*-schisandrin, and schisandrin C, respectively) was confirmed with standards by co-chromatography and by comparison of the LC-DAD data and LC-ESI-MS spectra with literature reports (Huang et al. 2007, 2008; Yang et al. 2011).

**Table 1:** Dibenzocyclooctadiene lignans identified in methanol extracts of *Schisandra chinensis* in vitro cultured biomass

No.^a^	t_R_ (min)	Compound^b^	λ_max_ (nm)^c^	LC-ESI-MS ions (*m/z*)^de^
1	3.78	**Schisandrin (Schisandrol A)**	213, 250, 280sh	433 [M + H]^+^, **415** [M + H – H_2_O]^+^, 400 [M + H – H_2_O – CH_3_]^+^, 384 [M + H – H_2_O – OCH_3_]^+^, 373 [M + H – H_2_O – C_3_H_6_]^+^, 369 [M + H – H_2_O – OCH_3_ – CH_3_]^+^, 359 [M + H – H_2_O – C_4_H_8_]^+^, 353 [M + H – H_2_O – OCH_3_ – OCH_3_]^+^
2	4.30	**Gomisin A (Schisandrol B)**	214, 254, 285sh	417 [M + H]^+^, **399** [M + H – H_2_O]^+^, 384 [M + H – H_2_O – CH_3_]^+^, 368 [M + H – H_2_O – OCH_3_]^+^, 357 [M + H – H_2_O – C_3_H_6_]^+^, 343 [M + H – H_2_O – C_4_H_8_]^+^, 337 [M + H – H_2_O – OCH_3_ – OCH_3_]^+^, 341 [M + H – H_2_O – CH_2_O – CO]^+^
3	5.50	Angeloygomisin H or Tigloylgomisin H^f^	218, 250sh, 285sh	523 [M + Na]^+^, 501 [M + H]^+^, 483 [M + H – H_2_O]^+^, **401** [M + H – C_5_H_8_O_2_]^+^, 383 [M + H – C_5_H_8_O_2_ – H_2_O]^+^, 357 [M + H – C_5_H_8_O_2_ – C_2_H_4_O]^+^
4	6.30	Angeloygomisin Q or Tigloylgomisin Q^f^	219, 255, 285sh	553 [M + Na]^+^, 531 [M + H]^+^, **431** [M + H – C_5_H_8_O_2_]^+^ , 389 [M + H – C_5_H_8_O_2_ – C_3_H_6_]^+^, 387 [M + H – C_5_H_8_O_2_ – C_2_H_4_O]^+^
5	7.00	**Gomisin G**	220, 255sh, 280sh	**415** [M + H – C_7_H_6_O_2_]^+^, 397 [M + H – C_7_H_6_O_2_ – H_2_O]^+^, 384 [M + H – C_7_H_6_O_2_ – CH_3_O]^+^, 373 [M + H – C_7_H_6_O_2_ – C_3_H_6_]^+^, 371 [M + H – C_7_H_6_O_2_ – C_2_H_4_O]^+^, 356 [M + H – C_7_H_6_O_2_ – C_2_H_4_O – CH_3_]^+^ , 343 [M + H – C_7_H_6_O_2_ – C_2_H_4_O – CO]^+^, 341 [M + H – C_7_H_6_O_2_ – C_2_H_4_O – CH_2_O]^+^
6	8.10	**Schisantherin A (Gomisin C)**	217, 255sh, 285sh	**415** [M + H – C_7_H_6_O_2_]^+^, 397 [M + H – C_7_H_6_O_2_ – H_2_O]^+^, 385 [M + H – C_7_H_6_O_2_ – CH_2_O]^+^, 373 [M + H – C_7_H_6_O_2_ – C_3_H_6_]^+^, 371 [M + H – C_7_H_6_O_2_ – C_2_H_4_O]^+^, 340 [M + H – C_7_H_6_O_2_ – C_2_H_4_O – OCH_3_]^+^
7	8.50	**Schisantherin B (Gomisin B)**	214, 255sh, 293sh	**415** [M + H – C_5_H_8_O_2_]^+^, 397 [M + H – C_5_H_8_O_2_ – H_2_O]^+^, 385 [M + H – C_5_H_8_O_2_ – CH_2_O]^+^, 373 [M + H – C_5_H_8_O_2_ – C_3_H_6_]^+^, 371 [M + H – C_5_H_8_O_2_ – C_2_H_4_O]^+^, 340 [M + H – C_5_H_8_O_2_ – C_2_H_4_O – OCH_3_]^+^
8	9.80	**Schisanhenol**	211, 250, 287	**403** [M + H]^+^, 371 [M + H – CH_3_OH]^+^, 372 [M + H – OCH_3_]^+^
9	17.40	**Deoxyschisandrin (Schisandrin A)**	217, 248, 285sh	**417** [M + H]^+^, 402 [M + H – CH_3_]^+^, 386 [M + H – OCH_3_]^+^ , 370 [M + H – C_2_H_7_O]^+^, 347 [M + H – C_5_H_10_]^+^, 332 [M + H – C_5_H_10_ – CH_3_]^+^, 316 [M + H – C_5_H_10_ – OCH_3_]^+^
10	19.10	Schisandrin B	219, 255, 280sh	**401** [M + H]^+^, 386 [M + H – CH_3_]^+^, 371 [M + H – CH_2_O]^+^, 331 [M + H – C_5_H_10_]^+^, 300 [M + H – C_5_H_10_ – CH_3_O]^+^
11	19.60	***γ*** **-Schisandrin**	217, 255, 285	**401** [M + H]^+^, 386 [M + H – CH_3_]^+^, 371 [M + H – CH_2_O]^+^, 331 [M + H – C_5_H_10_]^+^, 300 [M + H – C_5_H_10_ – OCH_3_]^+^
12	19.80	Benzoylgomisin P	219, 255sh, 285	537 [M + H]^+^, **415** [M + H – C_7_H_6_O_2_]^+^, 384 [M + H – C_7_H_6_O_2_ – OCH_3_]^+^, 373 [M + H – C_7_H_6_O_2_ – C_3_H_6_]^+^
13	20.70	**Schisandrin C**	215, 259, 281	**385** [M + H]^+^, 370 [M + H – CH_3_]^+^, 355 [M + H – CH_2_O]^+^, 315 [M + H – C_5_H_10_]^+^
14	21.30	Schisantherin D or 6-O-Benzoylgomisin O or Benzoylisogomisin O^f^	212, 255sh, 280sh	521 [M + H]^+^, **399** [M + H – C_7_H_6_O_2_]^+^, 384 [M + H – C_7_H_6_O_2_ – CH_3_]^+^, 381 [M + H – C_7_H_6_O_2_ – H_2_O]^+^, 369 [M + H – C_7_H_6_O_2_ – CH_2_O]^+^, 368 [M + H – C_7_H_6_O_2_ – OCH_3_]^+^

^a^Numbers correspond with peak labels in Fig.4

^b^Names in bold indicate compounds identified by co-chromatography with standards

^c^sh, shoulder

^d^Values in bold indicate base peak ion

^e^Proposed fragmentation patterns of compounds 3, 4, 10, 12, and 14 included as supplementary Figs. S1–S5

^f^Exact discrimination of isomers not possible under LC-MS analysis

Reference substances were not available for compounds 3, 4, 10, 12, and 14 which were identified based on the recorded LC-ESI-MS spectra. Compounds 3 and 4 gave the molecular ions at *m*/*z* 501 and 531, respectively, as well as fragment ions [M + H – 100]^+^ reflecting the loss of angeloyl or tigloyl moiety (*m*/*z* 401 and 431, respectively). Based on the observed fragmentation patterns (Figs. S1 and S2 in the Supplementary Material, and Table 1) and available literature data (Huang et al. 2007, 2008; Yang et al. 2011), the unknown constituents were tentatively identified as angeloylgomisin H or tigloylgomisin H (peak 3), and angeloylgomisin Q or tigloylgomisin Q (peak 4).

The LC-ESI-MS spectrum of compound 10 was virtually identical to that of *γ*-schisandrin (11) indicating that the two constituents are likely stereoisomers. The fragmentation patterns of both constituents (Fig. S3 in the Supplementary Material) reflected the cleavage of octadiene ring [M + H – 70]^+^ and subsequent loss of methoxy group [M + H – 70 – 31]^+^, as well as the loss of CH_3_ or CH_2_O at C-14 (fragment ions at [M + H – 15]^+^ and [M + H – 30]^+^, respectively) (Huang et al. 2007, 2008; Yang et al. 2011). Given the obtained results, peak 10 was attributed to schisandrin B.

The ESI-MS fragmentation pattern of compound 12 (Fig. S4 in the Supplementary Material) followed that of gomisin G (5) and schisantherin A (6), thus suggesting an isomeric structure. The fragment ion at *m*/*z* 415 corresponded to the loss of benzoic acid at C-6 (Huang et al. 2007). Consequently, 12 was tentatively identified as benzoylgomisin P (Ikeya et al. 1990).

The compound represented by peak 14 showed a similar ESI-MS fragmentation pattern (Fig. S5 in the Supplementary Material), characterized by the presence of molecular ion at *m/z* 521 and strong fragment ion at m/z 399 corresponding to the loss of benzoic acid ([M + H – 122]^+^). Given that the observed ESI-MS fragmentation mechanism of the above constituents was similar to that of schisantherin A (Huang et al. 2007), peak 14 was tentatively attributed to schisantherin D or one of its isomeric structures: 6-O-benzoylgomisin O and benzoylisogomisin O (Shi et al. 2009).

### Accumulation of dibenzocyclooctadiene lignans in shoot-differentiating callus from agar cultures

In the biomass extracts from the agar shoot-differentiating callus cultures, the presence of the main dibenzocyclooctadiene lignans was found (Table 2). A higher total content (237.86 mg/100 g DW) was obtained in the biomass harvested after 30 days than after 60 days of cultivation (185.61 mg/100 g DW). The dominant compounds were schisandrin (52.96 mg/100 g DW), deoxyschisandrin (30.27 mg/100 g DW), and gomisin A (25.93 mg/100 g DW). There were also significant amounts of the tentatively identified angeloyl-/tigloylgomisin Q (max. 51.24 mg/100 g DW), angeoyl-/tigloylgomisin H (max. 26.66 mg/100 g DW), and benzoylgomisin P (max. 17.50 mg/100 g DW).

**Table 2:** Contents (mg/100 g DW) of studied lignans in biomass extracts from different types of tested systems of cultivation of in vitro cultures of *Schisandra chinensis* maintained in the batch and fed batch mode

Lignans	Agar cultures		Stationary liquid cultures			Agitated cultures		
	Batch mode of cultivation		Batch mode of cultivation		Fed-batch mode of cultivation (60/30 days)	Batch mode of cultivation		Fed-batch mode of cultivation (60/30 days)
	30 days	60 days	30 days	60 days		30 days	60 days	
Schisandrin	52.96 ± 1.52	38.78 ± 2.45	65.62 ± 2.68	30.15 ± 4.54	31.36 ± 4.65	37.98 ± 0.75	34.90 ± 0.81	42.24 ± 5.55
Gomisin A	25.9 ± 1.863	20.70 ± 0.97	34.36 ± 1.24	13.04 ± 3.41	14.23 ± 0.75	24.37 ± 0.20	21.18 ± 1.21	27.66 ± 1.99
Gomisin G	1.44 ± 0.06	1.23 ± 0.03	1.64 ± 0.46	1.29 ± 0.42	0.86 ± 0.12	1.42 ± 0.06	1.42 ± 0.11	1.91 ± 0.29
Schisantherin A	0.420.03	0.21 ± 0.04	0.73 ± 0.07	0.63 ± 0.07	0.85 ± 0.06	3.05 ± 0.22	3.39 ± 0.13	2.97 ± 0.28
Schisantherin B	0.80 ± 0.06	0.69 ± 0.01	0.93 ± 0.16	0.48 ± 0.06	0.53 ± 0.05	3.37 ± 0.23	3.72 ± 0.13	5.70 ± 0.50
Schisanthenol	0.51 ± 0.05	0.41 ± 0.01	0.52 ± 2.47	0.12 ± 0.05	0.17 ± 0.04	0.96 ± 0.04	1.28 ± 0.02	1.06 ± 0.14
Deoxyschisandrin	30.27 ± 0.89	23.38 ± 0.69	43.65 ± 1.17	23.13 ± 3.70	26.57 ± 1.75	27.64 ± 1.49	27.36 ± 1.70	35.65 ± 1.60
γ-Schisandrin	8.04 ± 0.44	6.43 ± 0.16	10.06 ± 0.57	5.01 ± 1.33	5.32 ± 050	7.63 ± 1.16	8.34 ± 1.08	10.28 ± 0.79
Schisandrin C	2.35 ± 0.12	1.79 ± 0.08	4.25 ± 0.57	1.95 ± 0.75	2.12 ± 0.18	1.95 ± 0.19	2.16 ± 0.16	3.68 ± 0.22
Angeoyl-/tigloylgomisin H	26.66 ± 2.79	20.96 ± 1.55	24.26 ± 1.23	16.46 ± 1.77	14.70 ± 1.43	22.00 ± 1.59	21.59 ± 1.83	26.44 ± 2.35
Angeoyl-/tigloylgomisin Q	51.24 ± 2.72	42.31 ± 2.55	49.73 ± 1.52	34.20 ± 1.65	26.26 ± 1.17	34.58 ± 1.68	31.86 ± 1.34	44.25 ± 2.30
Schisandrin B	14.45 ± 0.27	11.17 ± 0.30	14.51 ± 0.24	14.01 ± 0.68	9.87 ± 0.24	13.56 ± 1.24	13.09 ± 1.42	20.70 ± 1.47
Benzoylgomisin P	17.50 ± 0.61	13.68 ± 0.68	18.74 ± 0.40	15.12 ± 0.44	3.46 ± 0.11	13.68 ± 1.33	12.54 ± 1.73	17.05 ± 3.08
Schisantherin D	5.31 ± 0.16	3.89 ± 0.18	5.65 ± 0.11	5.52 ± 0.62	5.83 ± 0.27	2.97 ± 0.10	2.94 ± 0.17	5.21 ± 0.60
Total content	237.86 ± 11.58	185.61 ± 9.70	274.65 ± 12.46	161.10 ± 19.49	142.12 ± 11.32	195.15 ± 10.28	185.77 ± 11.84	244.80 ± 21.16

Values are the means of four experiments ± SD

### Accumulation of dibenzocyclooctadiene lignans in shoots from stationary liquid cultures

In the biomass extracts from the stationary liquid cultures maintained in Magenta^TM^ vessels, fourteen schisandra lignans were found, as presented in Table 2. These compounds were not present in the tested media. The main metabolites were: schisandrin (65.62 mg/100 g DW), deoxyschisandrin (43.65 mg/100 g DW), and gomisin A (34.36 mg/100 g DW). There were also very high amounts of the tentatively identified: angeloyl-/tigloylgomisin Q (max. 49.73 mg/100 g DW), angeloyl-/tigloylgomisin H (max. 24.26 mg/100 g DW), and benzoylgomisin P (max. 18.74 mg/100 g DW). The highest amounts of schisandra lignans, both the maximum total amount (274.65 mg/100 g DW) and the amounts of each of the compounds, were obtained in the biomass grown in batch mode and harvested after 30 days; these amounts were considerably lower after 60 days (161.10 mg/100 g DW). In the fed-batch mode (60 days, with fresh medium supplementation on the 30th day of experiment), the obtained total amount of schisandra lignans was the lowest (142.12 mg/100 g DW).

### Accumulation of dibenzocyclooctadiene lignans in shoots from agitated cultures

In the extracts from the biomass from the agitated cultures harvested every 10 days in the batch mode of cultivation, we obtained varied amounts of all of the analyzed dibenzocyclooctadiene lignans. The dynamics of accumulation of these compounds are shown in Table 3. All of the estimated compounds were not confirmed in the extracts of the tested medium. The amounts of individual lignans in biomass extracts ranged from 0.7 to 84.75 mg/100 g DW. The quantitatively dominant compounds were schisandrin (max. 79.55 mg/100 g DW), gomisin A (max. 51.80 mg/100 g DW), and deoxyschisandrin (max. 47.84 mg/100 g DW). Very high amounts were also obtained of the tentatively identified angeloyl-/tigloylgomisin Q or (max. 84.75 mg/100 g DW) and of angeloyl-/tigloylgomisin H (max. 47.37 mg/100 g DW). Maximum individual contents and also total contents (406.14 mg/100 g DW) were obtained in the biomass extracts harvested after 10 days of cultivation. In the extracts from the biomass harvested after 30 and 60 days of cultivation, there were found high, only slightly different, amounts of schisandra lignans, which were 195.15 and 185.77 mg/100 g DW, respectively.

**Table 3:** Contents (mg/100 g DW) of studied dibenzocyclooctadiene lignans in biomass extracts from agitated shoot cultures of *Schisandra chinensis* during 60-day growth cycles. Values are the means of four experiments ± SD

Lignans	Days of growth cycles					
	10 days	20 days	30 days	40 days	50 days	60 days
Schisandrin	79.55 ± 3.85	60.68 ± 1.36	37.98 ± 0.75	32.47 ± 1.58	21.28 ± 1.33	34.90 ± 0.81
Gomisin A	51.80 ± 2.41	42.75 ± 0.97	24.37 ± 0.20	25.51 ± 0.16	12.69 ± 0.88	21.18 ± 1.21
Gomisin G	4.12 ± 0.22	2.82 ± 0.10	1.42 ± 0.06	1.47 ± 0.10	0.81 ± 0.02	1.42 ± 0.11
Schisantherin A	6.76 ± 0.46	5.40 ± 0.15	3.05 ± 0.22	3.14 ± 0.11	2.15 ± 0.19	3.39 ± 0.13
Schisantherin B	7.22 ± 0.48	5.81 ± 0.15	3.37 ± 0.23	3.46 ± 0.11	2.43 ± 0.20	3.72 ± 0.13
Schisanthenol	1.79 ± 0.12	1.43 ± 0.13	0.96 ± 0.04	1.45 ± 0.16	0.70 ± 0.04	1.28 ± 0.02
Deoxyschisandrin	47.84 ± 1.60	38.18 ± 1.28	27.64 ± 1.49	25.06 ± 1.13	18.89 ± 1.57	27.36 ± 1.70
γ-Schisandrin	14.52 ± 1.11	11.40 ± 1.10	7.63 ± 1.16	7.82 ± 1.15	5.09 ± 1.29	8.34 ± 1.08
Schisandrin C	4.63 ± 0.34	3.00 ± 0.40	1.95 ± 0.19	1.96 ± 0.08	1.11 ± 0.07	2.16 ± 0.16
Angeoyl-/tigloylgomisin H	47.37 ± 2.23	34.91 ± 1.68	22.00 ± 1.59	21.14 ± 1.23	13.35 ± 1.11	21.59 ± 1.83
Angeoyl-/tigloylgomisin Q	84.75 ± 2.10	59.14 ± 1.51	34.58 ± 1.68	30.51 ± 1.19	18.86 ± 1.44	31.86 ± 1.34
Schisandrin B	23.98 ± 0.17	18.87 ± 1.36	13.56 ± 1.24	14.56 ± 1.32	9.84 ± 1.31	13.09 ± 1.42
Benzoylgomisin P	26.54 ± 1.20	20.01 ± 1.22	13.68 ± 1.33	13.55 ± 1.20	9.57 ± 1.16	12.54 ± 1.73
Schizantherin D	5.25 ± 0.06	4.27 ± 0.04	2.97 ± 0.10	3.36 ± 0.05	2.42 ± 0.12	2.94 ± 0.17
Total content	406.14 ± 16.35	308.69 ± 11.45	195.15 ± 10.28	185.46 ± 9.87	119.19 ± 10.73	185.77 ± 11.84

In the experiment with the fed-batch mode of cultivation (60 days with fresh medium supplementation on the 30th day), higher amounts of the individual lignans were found. The quantitatively dominant compounds were schisandrin (max. 42.24 mg/100 g DW), gomisin A (max. 27.66 mg/100 g DW), and deoxyschisandrin (max. 35.65 mg/100 g DW). Considerable amounts were also obtained of the tentatively identified angeloyl-/tigloylgomisin Q (max. 44.25 mg/100 g DW) and of angeloyl-/tigloylgomisin H (max. 26.44 mg/100 g DW). The maximum total amount of the estimated compounds was high (244.80 mg/100 g DW) (Table 2).

### Accumulation of dibenzocyclooctadiene lignans in plant material

In the extracts from the leaves of the parent plant growing in vivo, a high total amount of the analyzed compounds was obtained—322.83 mg/100 g DW (Table 4). Quantitatively dominant were lignans other than those analyzed in the extracts from the biomass from in vitro cultures. The high amounts obtained were those of gomisin G (49.10 mg/100 g DW), deoxyschisandrin (41.01 mg/100 g DW), gomisin A (34.50 mg/100 g DW), and schisandrin (29.69 mg/100 g DW). There were also considerable amounts of the tentatively identified angeloyl-/tigloylgomisin H (max. 51.21 mg/100 g DW), and of schisandrin B (max. 21.84 mg/100 g DW).

**Table 4:** The maximum contents (mg/100 g DW ± SD) of estimated dibenzocyclooctadiene lignans in biomass extracts from in vitro cultures and in extracts from fruits and leaves of parent plant

Lignans	In vitro cultures			Plant material	
	Agar	Stationary liquid	Agitated	Leaves	Fruits
Schisandrin	52.96 ± 1.52	65.62 ± 2.68	42.24 ± 5.55	29.69 ± 1.42	132.39 ± 9.42
Gomisin A	25.9 ± 1.863	34.36 ± 1.24	27.66 ± 1.99	34.50 ± 2.16	109.40 ± 8.26
Gomisin G	1.44 ± 0.06	1.64 ± 0.46	1.91 ± 0.29	49.10 ± 2.89	46.06 ± 3.09
Schisantherin A	0.42 ± 0.03	0.85 ± 0.06	2.97 ± 0.28	25.86 ± 3.11	25.48 ± 2.50
Schisantherin B	0.80 ± 0.06	0.93 ± 0.16	5.70 ± 0.50	3.43 ± 0.32	4.73 ± 0.99
Schisanthenol	0.51 ± 0.05	0.52 ± 2.47	1.28 ± 0.02	2.66 ± 0.50	3.62 ± 0.42
Deoxyschisandrin	30.27 ± 0.89	43.65 ± 1.17	35.65 ± 1.60	41.01 ± 3.82	60.72 ± 5.20
γ-Schisandrin	8.04 ± 0.44	10.06 ± 0.57	10.28 ± 0.79	22.27 ± 1.99	66.50 ± 2.51
Schisandrin C	2.35 ± 0.12	4.25 ± 0.57	3.68 ± 0.22	10.91 ± 1.28	6.10 ± 1.03
Angeoyl-/tigloylgomisin H	26.66 ± 2.79	24.26 ± 1.23	26.44 ± 2.35	51.21 ± 5.82	161.88 ± 10.12
Angeoyl-/tigloylgomisin Q	51.24 ± 2.72	49.73 ± 1.52	44.25 ± 2.30	8.19 ± 1.01	52.34 ± 4.92
Schisandrin B	14.45 ± 0.27	14.51 ± 0.24	20.70 ± 1.47	21.84 ± 1.28	56.77 ± 3.01
Benzoylgomisin P	17.50 ± 0.61	18.74 ± 0.40	17.05 ± 3.08	14.38 ± 1.07	13.35 ± 0.39
Schisantherin D	5.31 ± 0.16	5.65 ± 0.27	5.21 ± 0.60	7.77 ± 0.39	15.22 ± 0.92
Total content	237.86 ± 11.58	274.65 ± 12.46	244.80 ± 21.16	322.83 ± 27.06	754.56 ± 52.78

In the extracts from the fruits, the main compounds were different from those in the leaves, namely, the tentatively identified angeloyl-/tigloylgomisin H (max. 161.88 mg/100 g DW), and schisandrin (132.39 mg/100 g DW), gomisin A (109.4 mg/100 g DW), *γ*-schisandrin (66.50 mg/100 g DW), and deoxyschisandrin (60.72 mg/100 g DW). The fruits were also found to contain high amounts of other tentatively identified lignans: angeloyl-/tigloylgomisin Q (max. 52.34 mg/100 g DW) and schisandrin B (max. 56.77 mg/100 g DW). The total amount of lignans in the fruits (754.56 mg/100 g DW) was 2.34 times higher than that in the leaves.

## Discussion

The increases in biomass in the agar culture cultivated in Magenta^TM^ vessels were relatively low. The increases in the biomass of the shoots cultivated in the same vessels in liquid medium were considerably higher. Many in vitro cultures grow better in liquid media, a representative example of which are liquid shoot cultures of three *Hypericum* cultivars (Kwiecień et al. 2015; Savio et al. 2012). Our experiments demonstrated that the established agitated shoot culture was characterized by a rather slow growth, requiring a 40-day period to go into the stationary phase. The long *lag* phase was probably caused by agitating, which hampered shoot growth during the first three weeks of the experiment (the phase of linear growth started just after the biomass had become immobilized in the growth vessel). The complete growth cycle until the dying phase lasted longer than in the average cell culture (Luczkiewicz and Glod 2005; Kokotkiewicz et al. 2015; Raj et al. 2015). Nevertheless, our work has proved that *Schisandra chinensis* shoots can be grown in an agitated system, which is a necessary step before further scale-up studies with bioreactors.

Apart from examining the influence of orbital agitating on shoot growth, both stationary and agitated liquid cultures were maintained under fed-batch conditions, and the results were compared with the experiments carried out in the batch mode. Fed-batch cultivation is considered a simple, yet effective way to improve the productivity of an in vitro system with respect to biomass and/or secondary metabolite yield (Georgiev et al. 2009). In the presented work, the addition of fresh medium on the 30th day of experiment produced positive effects, but only in the case of agitated cultures, confirming that medium mixing is essential to provide an effective mass transfer in *S. chinensis* in vitro cultures. In the case of stationary liquid cultures, the fed-batch cultivation slightly decreased the growth parameters in comparison with the batch mode, probably due to reduced oxygen transfer.

Given the obtained results, *S. chinensis* shoots are expected to grow well in air-agitated systems such as airlift and bubble column bioreactors. However, good results could probably also be achieved using temporary immersion systems which provided sufficient culture aeration and periodical medium mixing during the immersion phase (Steingroewer et al. 2013).

The results of phytochemical analyses confirmed the identical qualitative profile of lignans in all the in vitro culture systems analyzed, and also in the organs of parent plants.

As indicated in previous reports (Szopa et al. 2015; Szopa and Ekiert 2011, 2013, 2015), the HPLC analysis of schisandra lignans is often challenging since it involves working with multi-ingredient extracts, characterized by the presence of isomeric compounds with similar spectral properties. Nevertheless, the use of LC-MS techniques enabled to thoroughly investigate the composition of *S. chinensis* fruits with respect to lignan constituents (Huang et al. 2007, 2008; Shi et al. 2009).

In the present work, LC-MS was applied for the first time for the analysis of dibenzocyclooctadiene lignans in the biomass from *S. chinensis* in vitro cultures. The results indicate that the investigated cultures possess the ability to accumulate compounds typical for the parent plant. Besides, confirming the identity of the six constituents of *S. chinensis* in in vitro culture (schisandrin, gomisin A and G, deoxyschisandrin, schisantherin A, and *γ*-schisandrin) documented by us previously (Szopa et al. 2015; Szopa and Ekiert 2011, 2013, 2015), we additionally confirm, for the first time, the presence of shisanhenol, schisantherin B, and schisandrin C, and also of the tentatively identified compounds schisandrin B, benzoylgomisin P, angeloyl-/tigloylgomisin H, angeloyl-/tigloylgomisin Q, and schisantherin D (or one of its stereoisomers—benzoylgomisin O or benzoylisogomisin O) (Fig. 4 and Table 1). The substitution at C-6 position was reflected by the presence of fragmentation ions [M + H – 100]^+^ and [M + H – 122]^+^ which are indicative of angeloyl/tigloyl (2,4) and benzoyl derivatives (12,14), respectively. Most of the recorded spectra also showed the presence of [M + H – 15]^+^, [M + H – 18]^+^, [M + H – 30]^+^, [M + H – 31]^+^, or [M + H – 42]^+^ ions, reflecting a typical fragmentation pattern of dibenzocycloctadiene lignans. The individual compounds were identified by comparison of their ESI-MS spectra with those already reported (Huang et al. 2007, 2008; Shi et al. 2009). However, since dibenzocyclooctadiene lignan stereoisomers could not be differentiated using LC-MS techniques (Huang et al. 2007, 2008; Shi et al. 2009), the spectra recorded for compounds 3, 4, and 14 correspond to two or more isomeric structures (Table 1). Nevertheless, the obtained results provide a basis for further phytochemical studies on schisandra in vitro cultures.

The results of the quantitative analyses of schisandra lignans in the extracts from the tested types of culture systems clearly demonstrated the influence of the technique and the duration of cultivation on the growth and accumulation of secondary metabolites.

Comparing the amounts of lignans obtained in the extracts from the biomass in 30- and 60-day samples in all the types of culture, both agar and liquid, higher amounts of the compounds were obtained after 30 days of cultivation (Table 2). Although higher rates of biomass growth were obtained after 60 days, the results of the accumulation of lignans indicate that it is the 30-day duration of cultivation that can be nominated as optimal for the production of the analyzed compounds. Batch 30-day cultures are the ones that are usually performed in plant biotechnology laboratories; for many species, it is after this period that the accumulation of secondary metabolites is the greatest. In our previous experiments with *Ruta graveolens* and *R. graveolens* ssp. *divaricata* cultures, we had found maximum amounts of the analyzed furanocumarins and umbelliferone on day 28 and day 35 of the cultivation period, respectively (in stationary liquid cultures). By comparison, maximum amounts of this group of metabolites in agitated cultures of these plants were confirmed on day 42 and day 35 of the growth cycle, respectively (Ekiert et al. 2001, 2005; Ekiert and Czygan 2005).

For both types of liquid culture, stationary and agitated, cultivated in batch mode, the maximum total contents were found in extracts from the biomass harvested after 30 days of cultivation—195.15 mg/100 g DW and 274.65 mg/100 g DW, respectively. The maximum total content for agar cultures, 237.86 mg/100 g DW after 30 days, was 1.15 times lower than the maximum total content from stationary liquid cultures, but also 1.2 times higher than that in agitated cultures. This indicates the usefulness of both liquid systems for schisandra in vitro culture.

In the experiments with stationary liquid cultures maintained in Magenta^TM^ vessels and with agitated cultures, in addition to the standard cultivation techniques, the 30- and 60-day batch mode, it was decided to use the technique of fed-batch mode. The fed-batch mode of cultivation gave hope of obtaining higher amounts of secondary metabolites, with the high biomass growth rates preserved. In the case of stationary liquid cultures, the amount of the analyzed schisandra lignans obtained from the biomass grown for 60-days in fed-batch mode was only 1.18 times higher compared with their amount in the biomass grown for 60 days without supplementary feeding. However, the maximum total content obtained after 60-days in fed-batch mode was almost 2.4 times lower than in the biomass collected after 30 days of batch mode cultivation (Table 2). Despite the high rates of biomass growth obtained in this model, a 30-day culture cycle should be considered as an optimal duration of cultivation that is favorable to the production of lignans in a stationary liquid system. In the case of agitated cultures, the fed-batch mode proved effective. The usefulness of this mode was proven for agitated cultures, in which the highest increases in biomass growth were obtained, and at the same time the highest amounts of the analyzed metabolites, the max. total content, was 244.80 mg/100 g DW. This amount was 1.25 and 1.32 times higher than in the extracts from the biomass from agitated cultures harvested after 30 and 60 days of batch mode cultivation, respectively.

In the agitated cultures, there was a clear change in the dynamics of the accumulation of lignans. The highest amounts of the individual compounds were obtained in the extracts analyzed at the beginning of the experiment—after 10 days. On the following days, these amounts decreased. This dependence can be explained by the strong action of stress factors arising during the transfer of the cultures from the solid to the liquid medium in Erlenmeyer flasks, and the stress associated with mechanical agitating to which they were subjected. Normally, the biomass of in vitro plant cultures is harvested during the stationary growth phase, in which the accumulation of secondary metabolites is most often optimal. Due to the slow growth of *S. chinensis* cultures, the duration of the experiment was extended to 60 days. The biomass harvested after 60 days accumulated almost the same amount of lignans in comparison with 30-day samples (Tables 2 and 3). Based on these observations, the 30-day cultivation cycle can be nominated as optimal for the production of dibenzocyclooctadiene lignans in agitated cultures of *S. chinensis* cultivated in batch mode.

Liquid shoot culture systems of cultivation are the most attractive alternative compared to callus or cell suspension cultures, and are an essential element in carrying out experiments in large-scale bioreactors with forced flow of aeration or culture medium, as well as for the production of high-value plant compounds, for example, alkaloids (Berkov et al. 2009; Mitra et al. 1997), coumarins (Ekiert et al. 2001, 2005; Ekiert and Czygan 2005), and phenolic acids (Kwiecień et al. 2015). The complete elimination of agar, an expensive ingredient, in the medium has helped in a substantial cost reduction (5.2 times), which was calculated and reported earlier (Pati et al. 2011; Sandal et al. 2001).

The amount of the main schisandra lignan, schisandrin, was 2.21 times higher in biomass extracts (cultivated for 30 days in the stationary liquid system) than in the extracts from the leaves of the parent plant, analyzed for comparison, but 2.02 times lower than in the fruits. Also, the maximum amounts of the other main lignans, gomisin A and deoxyschisandrin, were comparable with their amounts in the leaves, but 3.18 and 1.39 times lower than in the fruits, respectively (Table 4).

The amounts of lignans obtained in the extracts from the biomass cultured in vitro were high, largely comparable to those obtained in the leaves of the parent plant. High levels of lignans are also determined by the high degree of organogenesis of the biomass studied (Charlwood et al. 1990). Our results suggest that the in vitro culture of *S. chinensis* can be a potential alternative source of lignans.

The study of the production of secondary metabolites in plants is of major interest in the areas of plant biotechnology and phytochemistry. The liquid cultures system developed in this work appears to be an efficient technique to obtain a high amount of biomass of *S. chinensis* in vitro capable of biosynthesis of unique dibenzocyclooctadiene lignans. In a study conducted at another centre, it has been shown that embryogenic callus of schisandra (growing in darkness) accumulates gomisin N as the main metabolite (the maximum concentration was 54.70 mg/100 g DW), and also *γ*-schisandrin (max. 13.90 mg/100 g DW), gomisin A (11.90 mg/100 g DW), and schisandrin (max. 0.90 mg/100 g DW) (Březinová et al. 2010). The presence of deoxyschisandrin, gomisin G, schisantherin A, schisandrin C, schisantherin B, and schisanthenol, and other tentatively identified dibenzocyclooctadiene lignans, which were detected under our study in significant quantities, had not been revealed by Březinová’s team. By contrast, callus cultures cultivated in MS medium by a Japanese team produce gomisin A (up to 0.05 g%) and gomisin F (up to 0.04 g%) as the only compounds and do not have the capacity for producing any of the other compounds analyzed under our study (Kohda et al. 2012). Therefore, our results of the analysis of lignans are very promising; special attention should be paid to the high amounts of schisandrin, gomisin A, and deoxyschisandrin. A valuable aspect of this work is also the qualitative and quantitative determinations of other lignans: schisandrin B, benzoylgomisin P, angeloyl-/tigloylgomisin H, angeloyl-/tigloylgomisin Q, and schisantherin D (or one of its stereoisomers—benzoylgomisin O or benzoylisogomisin O), by the LC-MS and LC-DAD methods, i.e., lignans that so far have not been described in any of the available scientific papers dealing with the accumulation of these metabolites in the biomass of in vitro cultures of *S. chinensis*.

Some other plant species are also known to accumulate considerable amounts of various valuable classes of secondary metabolites in shoot biomass cultivated in liquid media. This is the case, for example, with *Catharanthus roseus* shoot culture producing ajmalicine (Satdive et al. 2003), *Hypericum perforatum* shoot cultivars producing phenolic acids (Kwiecień et al. 2015), *R. graveolens* shoot culture accumulating furanocoumarins and umbelliferone (Ekiert et al. 2001), *Stevia rebaudiana* in respect of steviol glycoside biosynthesis (Bondarev et al. 2002), shoot culture of *Isopterix canariensis* accumulating cardenolides (Schaller and Kreis 2006), *Salvia officinaris* shoot culture accumulating carnosic acid, carnosol, and rosmarinic acid (Grzegorczyk and Wysokińska 2008), and also *Securinegra suffruticosa* microshoot culture accumulating indolizidine alkaloids (Raj et al. 2015).

The obtained results prove high usefulness of liquid culture techniques for conducting large-scale in vitro cultivation of *Schisandra chinensis*. Similar experiments designed to test the suitability of liquid cultures in terms of their growth profiles as well as the production of secondary metabolites are an essential element of preliminary studies before laboratory-scale in vitro cultures are replaced with cultivation on a large laboratory scale, for example, shoot cultures of *Securinegra suffruticosa* (Raj et al. 2015; Kokotkiewicz et al. 2015), suspension cultures of *Cyclopia subternata* (Kokotkiewicz et al. 2013), or hairy roots of *Hyoscyamus niger* (Jaremicz et al. 2014).

This is the first report which documents the potential usefulness of *S. chinensis* shoot cultures cultivated in liquid systems for practical purposes. In the present work, two liquid shoot culture systems (stationary and agitated) of *S. chinensis* were established and evaluated for the production of dibenzocyclooctadiene lignans. The presence of fourteen metabolites in biomass extracts from agar culture, stationary and agitated liquid cultures, and in organs of the parent plant (leaves and fruits) was confirmed by the HPLC-DAD and LC-DAD-ESI-MS methods. The major outcome of the study is the finding that the investigated biomass is suitable for cultivation in liquid systems. We put forward our *S. chinensis* in vitro cultures as a good model for further studies on the accumulation of lignans, particularly in large-scale installations (bioreactors). Shoots of *S. chinensis* cultivated in liquid systems are a rich source of therapeutically important lignans, especially schisandrin, gomisin B, and deoxyschisandrin, whose amounts are comparable with the levels of these compounds in the leaves of the parent plant. Our results, compared to those obtained by other research centers, are very promising and encourage further studies. The developed liquid systems could be considered as a new, potential solution for the production of schisandra lignans.

## Electronic supplementary material

Below is the link to the electronic supplementary material.

ESM 1 (PDF 361 kb)
