# Supplementary material for: Accumulation of dibenzocyclooctadiene lignans in agar cultures and in stationary and agitated liquid cultures of Schisandra chinensis (Turcz.) Baill
Source: Appl Microbiol Biotechnol. 2015 Dec 21;100:3965–77. doi: 10.1007/s00253-015-7230-9 (PMC4824827; doi:10.1007/s00253-015-7230-9)
Supplement: Supplementary file 1 — (PDF 361 kb) [file 253_2015_7230_MOESM1_ESM.pdf]

Applied Microbiology and Biotechnology

Accumulation of dibenzocyclooctadiene lignans in agar cultures and in stationary and agitated liquid cultures of *Schisandra chinensis* (Turcz.) Baill.

Agnieszka Szopa[1], Adam Kokotkiewicz[2], Urszula Marzec-Wróblewska[3], Adam Bucinski[3], Maria Luczkiewicz[2], Halina Ekiert[1]*

[1]Chair and Department of Pharmaceutical Botany, Jagiellonian University, Collegium Medicum, ul. Medyczna 9, 30-688 Kraków, Poland

[2]Chair and Department of Pharmacognosy, Faculty of Pharmacy, Medical University of Gdansk, al. gen. J. Hallera 107, 80-416 Gdańsk, Poland

[3]Department of Biopharmacy, Faculty of Pharmacy, Ludwik Rydygier Collegium Medicum in Bydgoszcz, Nicolaus Copernicus University in Toruń, ul. dr A. Jurasza 2, 85-089 Bydgoszcz, Poland

*Corresponding author:
phone +48 12 620 54 30, fax +48 620 54 40, e-mail: a.szopa@uj.edu.pl

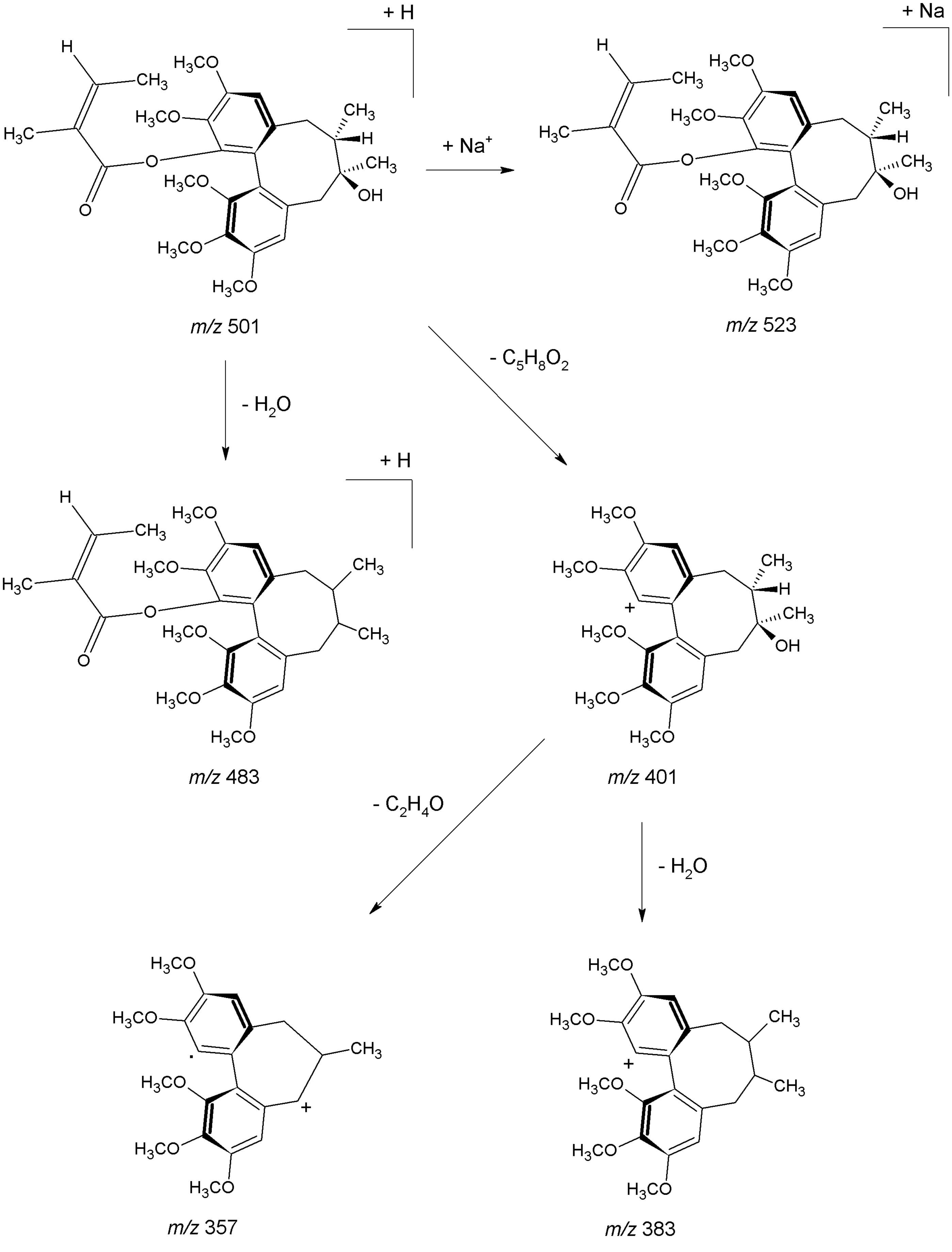

Fig. S1 A proposed fragmentation pattern of Angeloylgomisin H (**3**)

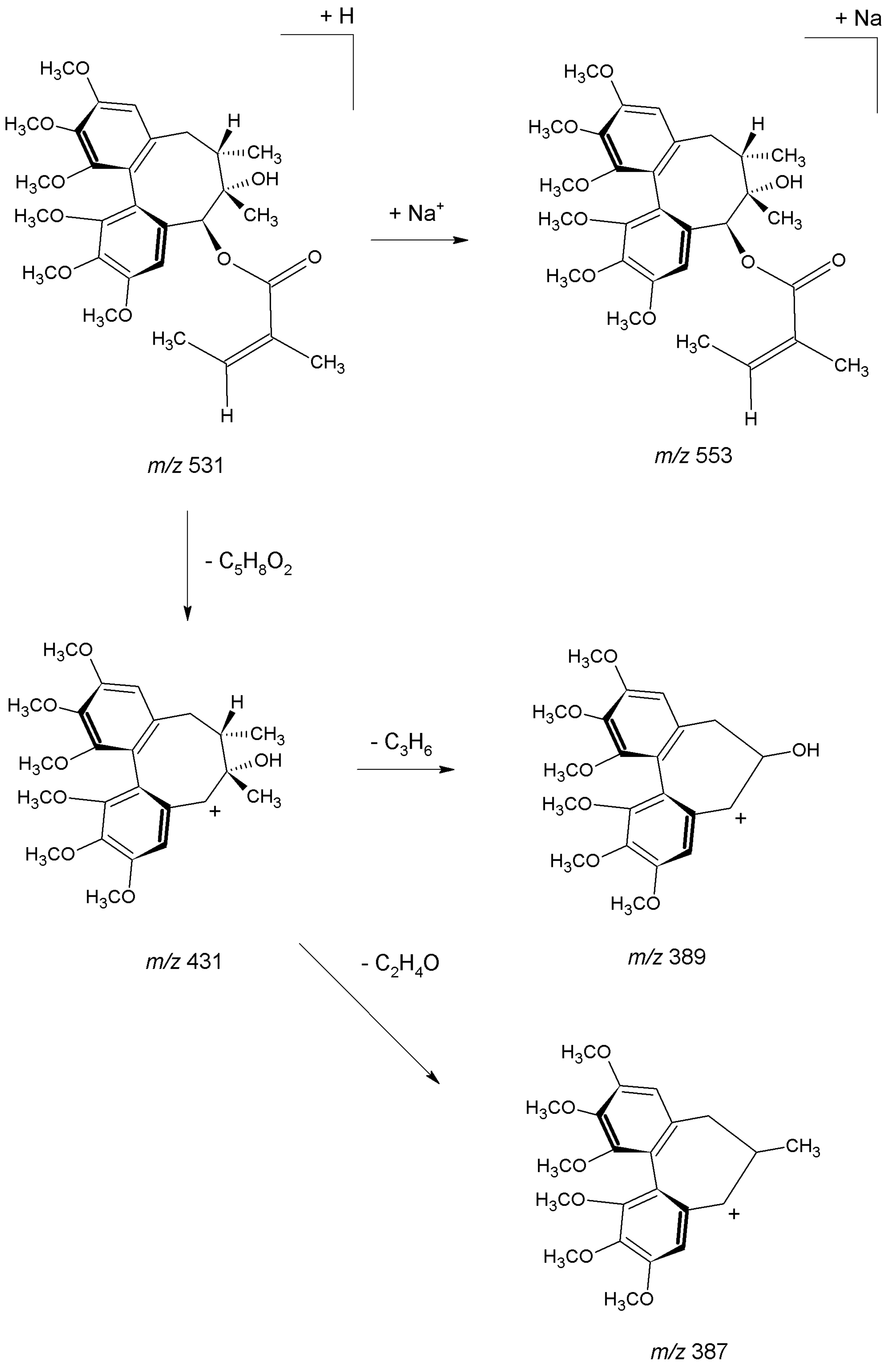

Fig. S2 A proposed fragmentation pattern of Angeloylgomisin Q (**4**)

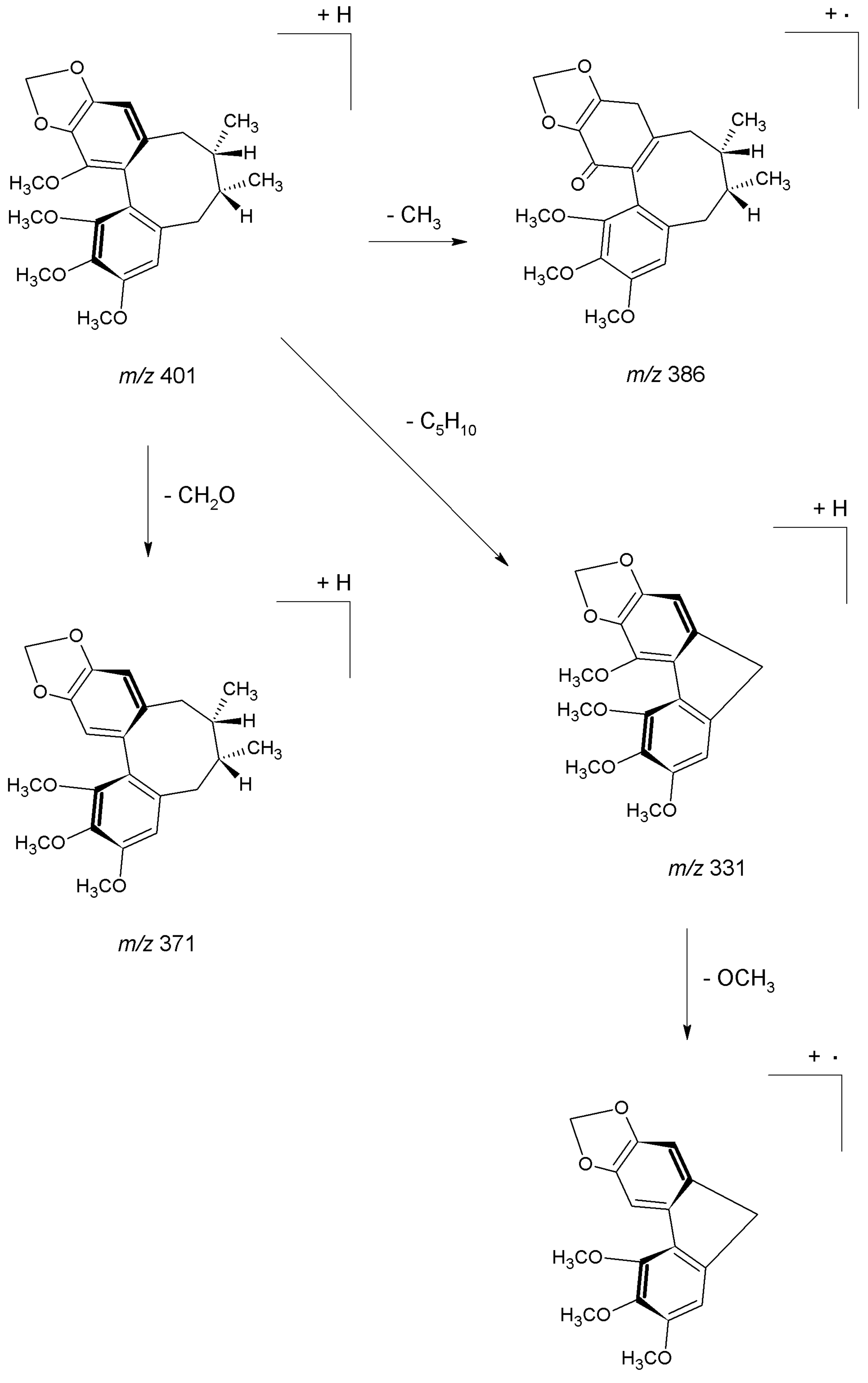

Fig. S3 A proposed fragmentation pattern of Schisandrin B (**10**)

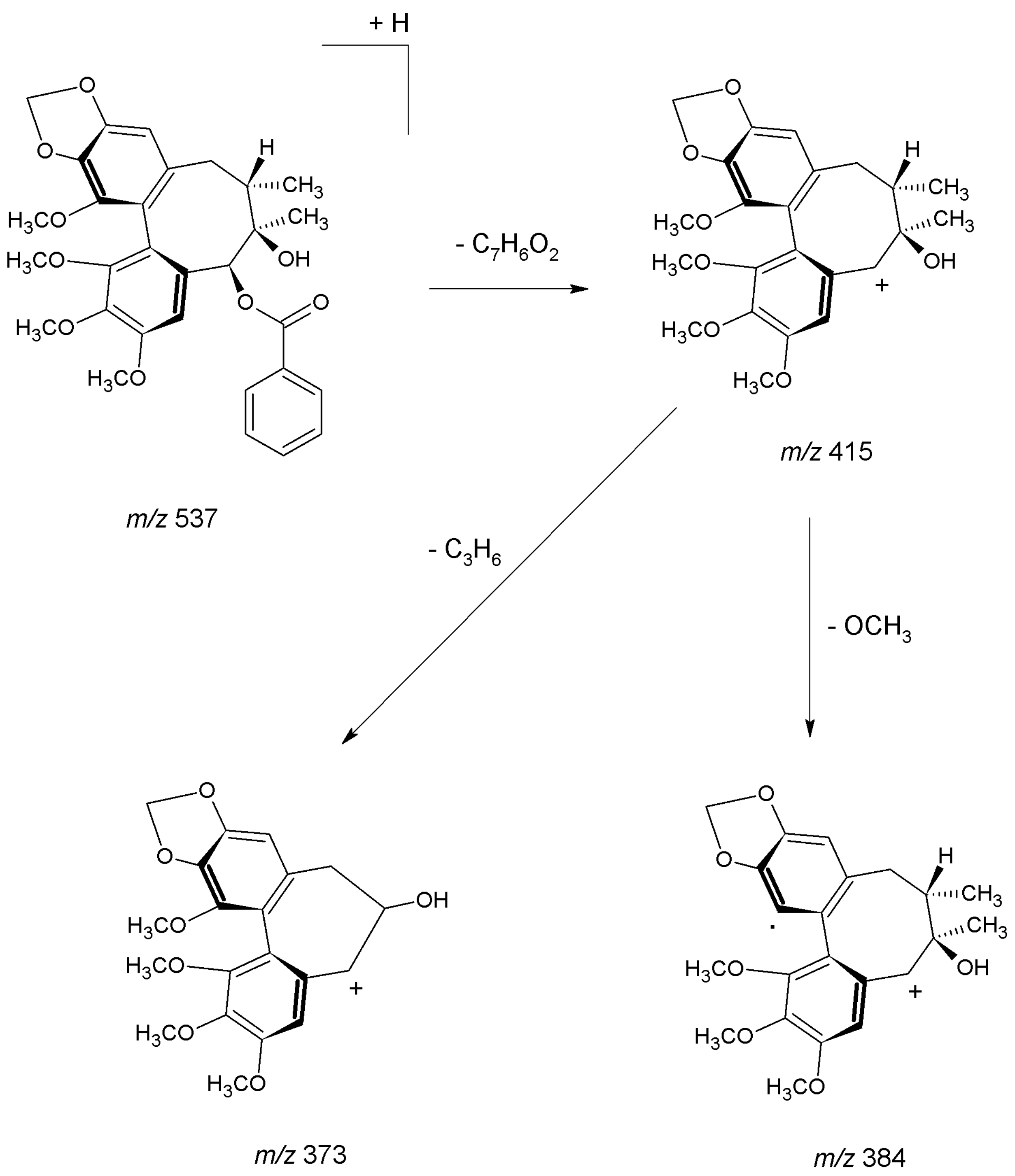

Fig. S4 A proposed fragmentation pattern of Benzoylgomisin P (**12**)

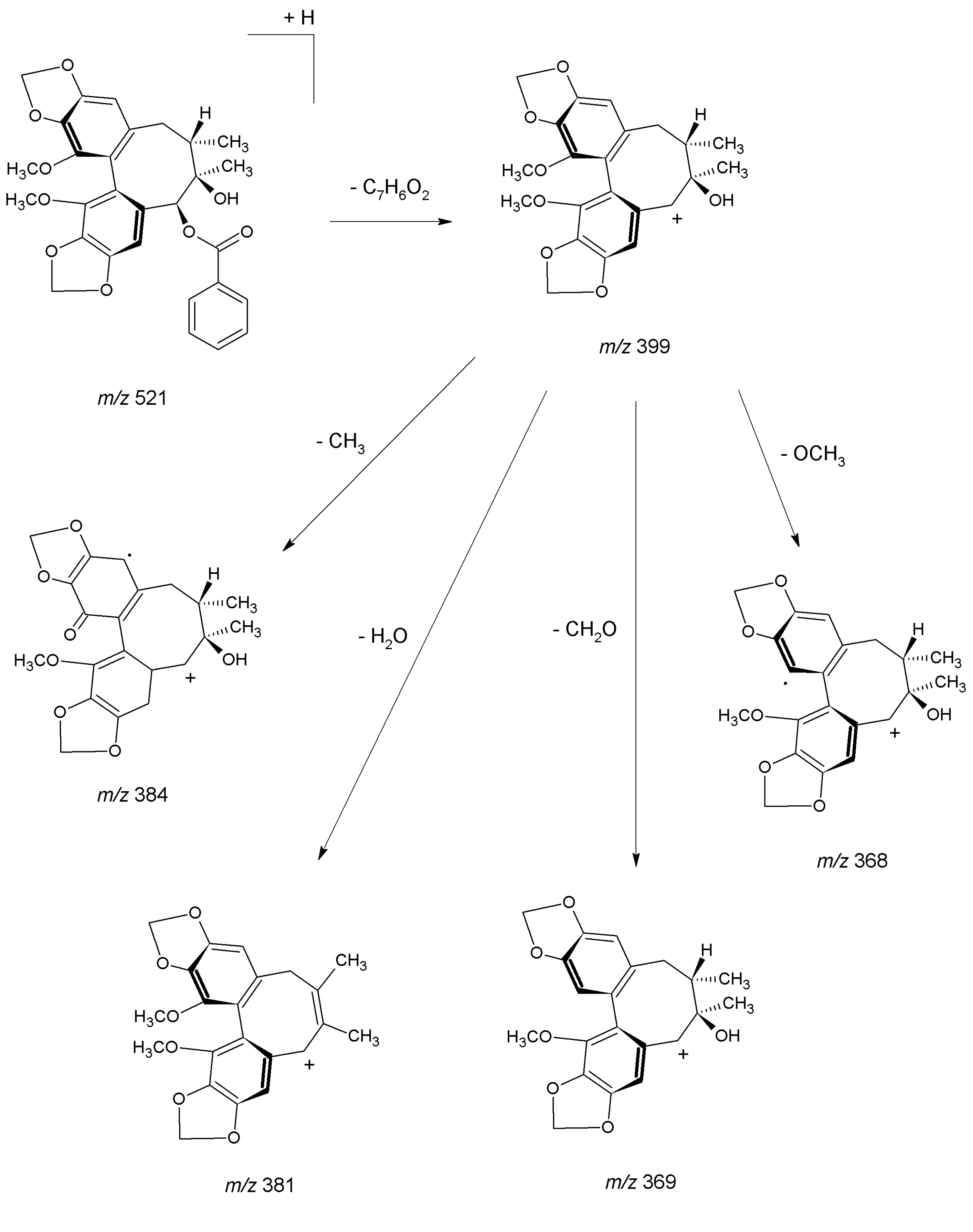

Fig. S5 A proposed fragmentation pattern of Schisantherin D (**14**)
